# Associations between specific volatile organic chemical exposures and cardiovascular disease risks: insights from NHANES

**DOI:** 10.3389/fpubh.2024.1378444

**Published:** 2024-05-23

**Authors:** Shaojie Han, Min Xie, Siyuan Cheng, Yuchen Han, Panpan Li, Jun Guo

**Affiliations:** ^1^The First Clinical Medical College, Jinan University, Guangzhou, China; ^2^Department of Cardiology, The First Affiliated Hospital of Jinan University, Guangzhou, China; ^3^Department of Cardiology, Seventh People’s Hospital of Chengdu, Chengdu, China

**Keywords:** volatile organic chemicals, cardiovascular diseases, environment pollution, Bayesian kernel machine regression, NHANES

## Abstract

**Introduction:**

An increasing body of research has demonstrated a correlation between pollutants from the environment and the development of cardiovascular diseases (CVD). However, the impact of volatile organic chemicals (VOC) on CVD remains unknown and needs further investigation.

**Objectives:**

This study assessed whether exposure to VOC was associated with CVD in the general population.

**Methods:**

A cross-sectional analysis was conducted utilizing data from five survey cycles (2005–2006, 2011–2012, 2013–2014, 2015–2016, and 2017–2018) of the National Health and Nutrition Examination Survey (NHANES) program. We analyzed the association between urinary VOC metabolites (VOCs) and participants by multiple logistic regression models, further Bayesian Kernel Machine Regression (BKMR) models and Weighted Quantile Sum (WQS) regression were performed for mixture exposure analysis.

**Results:**

Total VOCs were found to be positively linked with CVD in multivariable-adjusted models (p for trend = 0.025), independent of established CVD risk variables, such as hypertension, diabetes, drinking and smoking, and total cholesterol levels. Compared with the reference quartile of total VOCs levels, the multivariable-adjusted odds ratios in increasing quartiles were 1.01 [95% confidence interval (CI): 0.78–1.31], 1.26 (95% CI: 1.05–1.21) and 1.75 (95% CI: 1.36–1.64) for total CVD. Similar positive associations were found when considering individual VOCs, including AAMA, CEMA, CYMA, 2HPMA, 3HPMA, IPM3 and MHBMA3 (acrolein, acrylamide, acrylonitrile, propylene oxide, isoprene, and 1,3-butadiene). In BKMR analysis, the overall effect of a mixture is significantly related to VOCs when all chemicals reach or exceed the 75th percentile. Moreover, in the WQS models, the most influential VOCs were found to be CEMA (40.30%), DHBMA (21.00%), and AMCC (19.70%).

**Conclusion:**

The results of our study indicated that VOC was all found to have a significant association with CVD when comparing results from different models. These findings hold significant potential for public health implications and offer valuable insights for future research directions.

## 1 Introduction

Volatile organic compounds (VOC) are typical organic air pollutants that can come from both natural and industrial sources (1). VOC are present in both indoor and outdoor air due to factors such as cooking, cleaning, incense burning, smokey air, occupational gas exposure, industrial production processes, everyday items, and traffic pollution (2). The widespread presence of VOC in the air makes exposure to the general population easier and more common when compared to other pollutants that people are exposed to in particular environments. The exposure approach primarily entails lung inhalation, gastrointestinal absorption, and cutaneous infiltration (3). Researchers have used a range of methods to monitor human exposure to VOC, including conventional methods of evaluation and the collection of a person’s sweat, urine, breath, and blood (4).

Cardiovascular disease (CVD) continues to be the largest cause of adult mortality in the United States, despite improvements in prevention, diagnosis, and treatment (5). An increasing body of evidence suggests that exposure to environmental contaminants may be associated with an elevated risk of CVD (6). For instance, it has been observed that the presence of pyrethroid insecticides, polyfluoroalkyl compounds, and polycyclic aromatic hydrocarbons is positively correlated with the occurrence of CVD among the United States population. According to the World Health Organization (WHO), the global incidence of stroke and heart disease-related mortality is estimated to be 1.4 million and 2.4 million, respectively, on an annual basis, attributable to exposure to air pollution (7).

Meanwhile, numerous studies have documented the possible influence of exposure to VOC on many risk factors associated with CVD, including obesity, systemic inflammation, endothelial damage, and biomarkers indicative of oxidative stress (8–11). The findings of a randomized crossover intervention study suggest that persons with a higher body mass index (BMI) may be at an increased risk for CVD when exposed to VOC (12). Several studies have also demonstrated an association between exposure to VOC and vascular dysfunction or endothelial dysfunction (7, 9). Research conducted on animals has shown evidence that exposure to VOC (acrylamide) can have detrimental effects on glucose regulation, impair β-cell function, and decrease the size of the aorta artery. Additionally, VOC (acrylamide) exposure can reduce acetylcholine-mediated endothelial-dependent arterial diastolic responses (13, 14). Other studies have documented that short-term exposure to VOC is associated with adverse health effects such as renal impairment, liver damage, leukemia, and chromosomal aberrations (15–17). These mechanisms may highly overlap with the development of CVD.

However, few large-scale studies have directly reported that VOC exposure is closely related to CVD. Therefore, our study aims to thoroughly assess 16 major VOC metabolites (VOCs) in urine and establish the relationship between them and CVD, including heart attack, congestive heart failure, angina, coronary heart disease, and stroke.

## 2 Materials and methods

### 2.1 Study population

The National Health and Nutrition Examination Study (NHANES) is a comprehensive study performed biennially by the Centers for Disease Control and Prevention (CDC) to gather data on the health and nutritional status of the non-institutionalized population in the United States. The NHANES website^1^ provides comprehensive access to the survey data and methodological information. The NHANES procedures received approval from the Institutional Review Board (IRB) of NAHNES and the Research Ethics Review Board (ERB) of NCHS. In our study, no external IRB or ethical approval was needed beyond NHANES IRB/ERB approval. We constructed weights for combined survey cycles of NHANES 2005–2006, 2011–2012, 2013–2014, 2015–2016, and 2017–2018 according to the NHANES analytical guidelines. In the present investigation, further external IRB or ethical approval was not deemed necessary. In this work, weights were established for the combined NHANES survey cycles by the NHANES analytical criteria.

### 2.2 Measure of urine VOC metabolites

According to earlier investigations, ultra-performance liquid chromatography combined with electrospray tandem mass spectrometry (UPLC-ESI/MSMS) was used to quantitatively identify VOCs in human urine (18). Chromatographic separation was carried out using an Acquity UPLC® HSS T3 (Part No. 186003540, 1.8 m x 2.1 mm x 150 mm, Waters Inc.) column and mobile phases of 15 mM ammonium acetate and acetonitrile. On the website,^2^ you may obtain more information on the laboratory technique for detecting urine VOCs.

### 2.3 Assessment of CVD outcomes

Total CVD outcomes include any congestive heart failure, coronary heart disease, angina, heart attack, or stroke by positive self-reported medical diagnoses. In this study, a standardized medical condition questionnaire was administered as part of the personal interview process. All participants were queried on whether a medical practitioner or other healthcare professional had ever provided them with a diagnosis of congestive heart failure, coronary disease, heart disease, angina, heart attack, or stroke.

### 2.4 Covariates and CVD risk factors

The demographic questionnaire provided us with data on age, gender (male/female), race/ethnicity (non-Hispanic white, non-Hispanic black, Mexican American, other Hispanic, and other), family poverty income ratio (PIR), marital status, and educational levels (less than 9th grade, high school graduate, some college or AA degree, college graduate or above). Data about the levels of physical activity (never, moderate, and vigorous), alcohol consumption (having consumed a minimum of 12 alcoholic beverages within a year), and smoking habits (having smoked over 100 cigarettes in one’s lifetime) were collected from the surveys on physical activity, alcohol usage, and cigarette smoking. The BMI data for each participant was extracted from the examination records and was determined by dividing the weight of each participant by the square of their height. Additional risk factors for CVD, namely, LDL-cholesterol, total triglyceride, total cholesterol, HDL-cholesterol, C-reactive protein (CRP), glycosylated hemoglobin (HbA1c), insulin, and fasting plasma glucose were assessed in serum through laboratory techniques. The diagnosis of hypertension was made when a systolic blood pressure exceeding 140 mmHg or a diastolic blood pressure over 90 mmHg was seen. The diagnosis of diabetes was determined based on the data obtained from the questionnaire, the presence of a fasting plasma glucose level equal to or more than 7.0 mmol/L, or the use of antidiabetic medications. The data about urine creatinine and urine albumin were obtained from the laboratory test data of the NHANES study.

### 2.5 Statistical analysis

In this cross-sectional investigation, a correlation analysis was conducted to examine the relationship between exposure to VOCs in urine and CVD. The comparative analysis of baseline data between subgroups with CVD and non-CVD was conducted using the chi-square test for categorical data and the Kruskal-Wallis test for quantitative data. Subsequently, three quantile logistic regression models were developed, each incorporating distinct variables, to examine the relationship between urinary VOCs and CVD. The quantile logistic regression study involves categorizing the concentration of VOCs into four intervals based on their concentration levels, ranging from low to high. Model 1 was adjusted for baseline age, gender, race, and NHANES cycles. Furthermore, in model 2, adjustments were made for the following variables: BMI, education level (less than 9th grade, high school graduate, some college or AA degree, college graduate or above) marital status (categorized as married, separated, widowed, divorced, never married, and others), family PIR, smoking status, drinking status, hypertension, diabetes, and physical activity level. In model 3, adjustments were made for kidney function, namely urine creatinine and urine albumin, as well as total cholesterol, in addition to the variables mentioned above. Our used subgroup analyses to investigate the relationships between VOCs and CVD, such as heart attack, congestive heart failure, angina, coronary heart disease, and stroke. Then, stratified models were employed to examine the potential relationships between exposure to VOCs. Weighted quantile sum (WQS) regression was employed to investigate the comprehensive impacts of VOCs on CVD, as it demonstrated effectiveness in delineating the composition of environmental mixtures. We extended our inquiry to examine the collective impacts of mixed VOCs exposures using Bayesian kernel machine regression (BKMR) models.

The study involved the computation of Spearman correlation coefficients to assess the connection between urinary VOCs levels. The adjustment of sample weight was applied to the baseline data and comparison findings of VOCs between groups with CVD and non-CVD, as a consequence of including numerous survey cycles. Statistical significance was defined as a *p* value <0.05. The R software (version 4.3) was used for all statistical analyses.

## 3 Results

### 3.1 Sample characteristics

**Figure 1:**
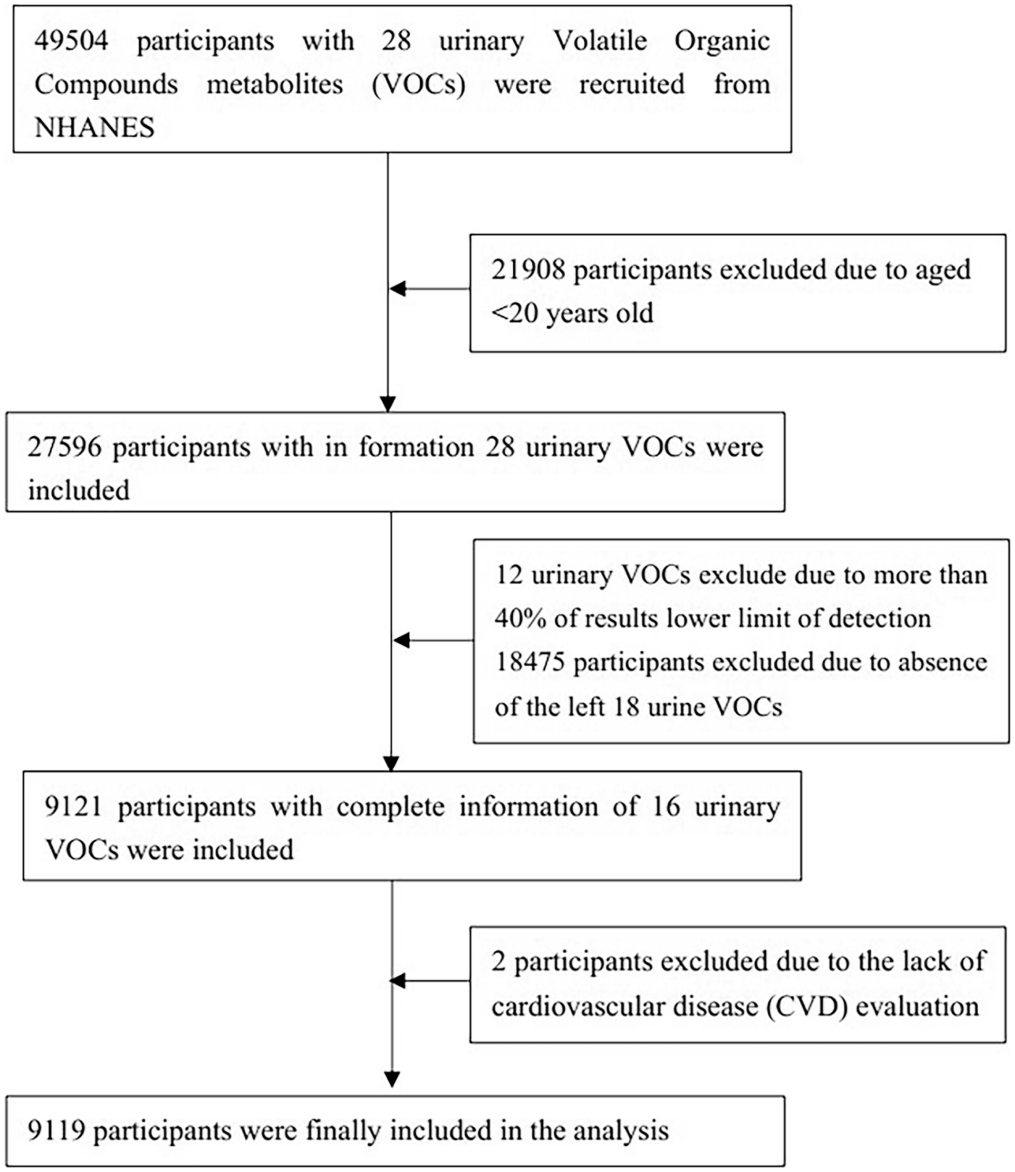
Flowchart of the study.

As seen in Figure 1, a preliminary inclusion of 49,504 people with data on 28 urine VOCs was made. A total of 21,908 individuals were eliminated from the study owing to being under the age of 20. Twelve VOCs present in urine were excluded from the analysis due to a high percentage (>40%) of values falling below the lower limit of detection (LLOD). After excluding participants who did not exhibit measurable VOCs, the remaining sample size consisted of 9,121 participants. Two individuals were eliminated because they did not undergo examination for CVD. Finally, 9,119 participants were analyzed in our study. Supplementaary Table S1 shows relative parent compounds to VOCs detected in this study. Table 1 presents the basic characteristics of the research population. The individuals were divided into two subgroups: those with CVD and those without CVD. A notable difference was seen between the two cohorts with regard to age, gender, marital status, race, family PIR, education level, BMI, smoking habits, alcohol consumption, presence of hypertension, diabetes, and levels of physical activity.

**Table 1:** Characteristics of the study population by CVD status.

Characteristics	CVD(931)	non-CVD(8187)	*p*-value
Age (years, mean ± SD)	66.14 ± 12.69	47.08 ± 17.19	<0.001
Male, *n* (%)	546 (58.61)	3,925 (47.94)	<0.001
Race origin, *n* (%)			<0.001
Mexican American	88 (9.45)	1,314 (16.05)	
Non-Hispanic White	474 (50.91)	3,140 (38.35)	
Non-Hispanic Black	229 (24.60)	1814 (22.16)	
Other Hispanic	70 (7.52)	724 (8.84)	
Other races	70 (7.52)	1,195 (14.60)	
Education level, *n* (%)			<0.001
Less than 9th grade	143 (15.36)	826 (10.09)	
9-11th grade	151 (16.22)	1,028 (12.56)	
High school graduate	252 (27.07)	1791 (21.88)	
Some college or AA degree	240 (25.78)	2,480 (30.29)	
College graduate or above	144 (15.47)	2056 (25.11)	
Refused/missing	1 (0.10)	6 (0.07)	
Marital status, *n* (%)			<0.001
Married	472 (50.70)	4,273 (52.19)	
Widowed	169 (18.15)	499 (6.10)	
Divorced	155 (16.65)	793 (9.69)	
Separated	31 (3.33)	254 (3.10)	
Never married	65 (6.98)	1,634 (19.95)	
Others	39 (4.19)	734 (8.97)	
Family PIR, (mean ± SD)	2.13 ± 1.45	2.60 ± 1.64	<0.001
BMI, (kg/m^2^, mean ± SD)	30.08 ± 7.09	29.02 ± 6.89	
Smoking^a^, *n* (%)			<0.001
Yes	570 (61.22)	3,456 (42.21)	
No	361 (38.78)	4,731 (57.79)	
Consume alcohol^b^, *n* (%)			0.003
Yes	543 (58.32)	5,000 (61.07)	
No	320 (34.37)	2,368 (28.92)	
Missing	68 (7.31)	819 (10.01)	
Hypertension, *n* (%)			<0.001
Yes	692 (74.33)	2,549 (31.13)	
No	239 (25.67)	5,638 (68.87)	
Diabetes, *n* (%)			<0.001
Yes	310 (33.30)	873 (10.66)	
No	621 (0.670)	7,314 (89.34)	
Physical activity, *n* (%)			<0.001
Never	452 (48.55)	2,695 (32.92)	
Moderate	316 (33.94)	2,421 (29.57)	
Vigorous	163 (17.51)	3,071 (37.51)	
Urine albumin (mg/L, mean ± SD)	140.93 ± 623.51	37.08 ± 267.44	<0.001
Urine creatinine(umol/L, mean ± SD)	9.92 ± 6.11	10.63 ± 6.93	0.001

a Smoking: having smoked over 100 cigarettes in one’s lifetime.

b Consume alcohol: having consumed a minimum of 12 alcoholic beverages within a year.

CVD, cardiovascular disease; PIR, family poverty income ratio; BMI, body mass index.

### 3.2 Profiles of urinary VOC metabolites (VOCs) in general people

**Figure 2:**
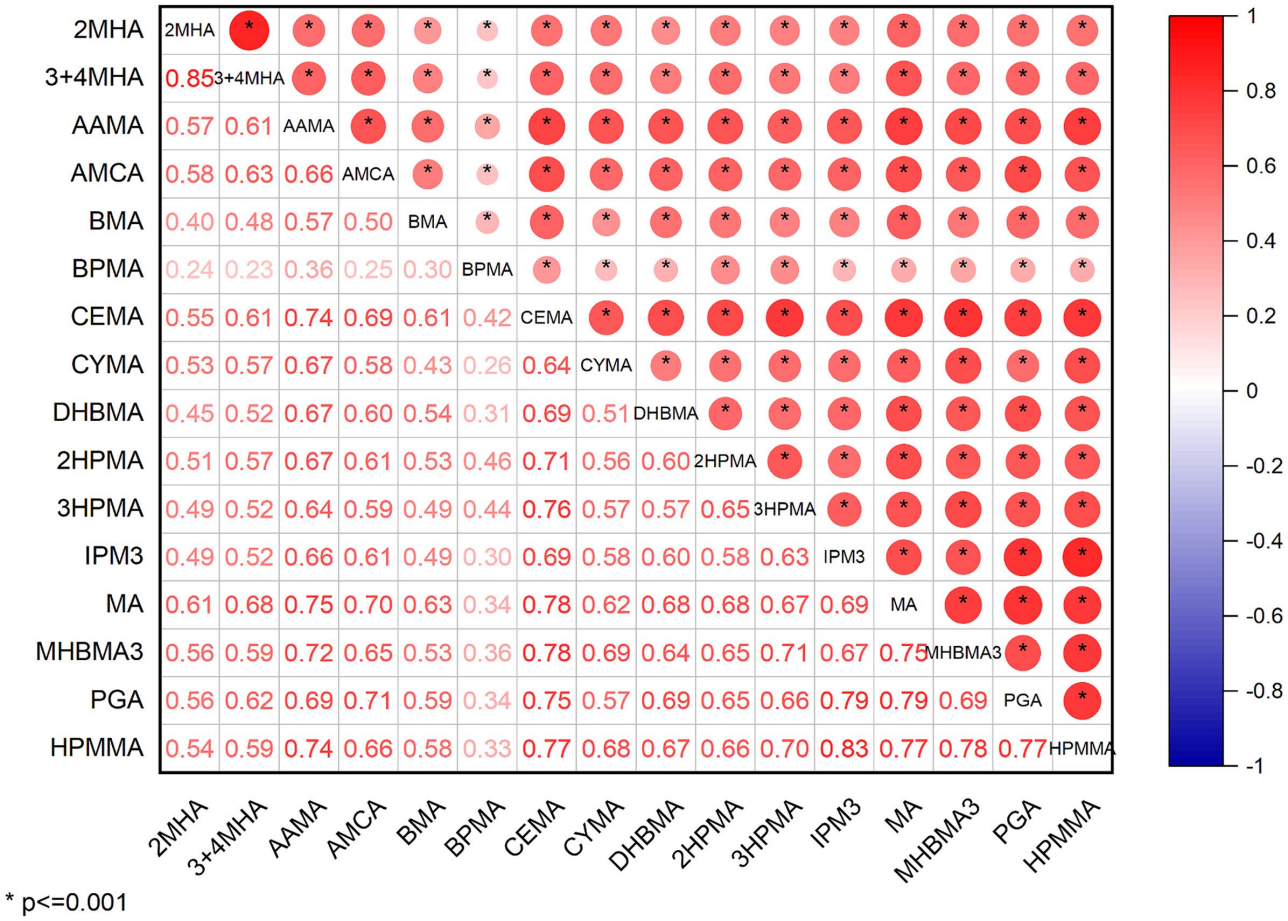
Spearman’s rank correlation coefficients of VOCs measured in NHANES.

The distribution of total and individual urinary VOCs levels in the entire study group, as well as in the groups diagnosed with and without CVD, is presented in Table 2. In our study, we investigated 15 VOCs. The results showed that 9 urinary VOCs were discovered in a majority of participants (98.5%) in the eligible survey cycles: 3MHA + 4MHA, AAMA, AMCC, BMA, CEMA, DHBMA, 3HPMA, MA, and HPMMA. The levels of all urinary VOCs shown in Supplementary Table S3 similarly demonstrated that some concentrations of VOCs were significantly elevated in the CVD group by sampling weight. Nearly all of the chosen urinary VOCs showed positive and substantial associations (*p* < 0.01). Detailed results can be viewed in Supplementary Table S1. Strong relationships were discovered between 2MHA and 3MHA + 4MHA (*r* = 0.876), HPMMA and 3HPMA (*r* = 0.824), IPM3 and MHBMA3 (*r* = 0.818), MHBMA3 and IPM3 (*r* = 0.852), and HPMMA and MHBMA3 (*r* = 0.856), as shown in Figure 2.

**Table 2:** The distribution levels of urinary VOCs in study population with and without CVD in NHANES.

VOCs	>LLOD(%)	Total study population		Study population with CVD		Study population without CVD	
		Median (IQR)	5th–95th percentile	Median (IQR)	5th–95th percentile	Median (IQR)	5th–95th percentile
Total VOCs	-	2088.55 (1186.60–3534.76)	455.08–9709.37	2237.55 (1342.90–3876.78)	512.09–10408.26	2073.27 (1165.43–3482.45)	449.78–9605.85
2MHA	92.36	28.90 (13.00–73.80)	3.54–226.00	27.80 (12.60–73.60)	3.54–219.40	29.00 (13.10–73.90)	3.54–227.00
3MHA + 4MHA	99.23	188.00 (80.85–492.00)	26.15–1620.00	197.00 (89.05–499.00)	26.85–1595.00	187.00 (80.00–490.00)	26.10–1630.00
AAMA	99.57	51.50 (69.00–295.00)	8.90–291.00	50.30 (26.10–97.90)	8.65–246.00	51.60 (26.25–103.00)	8.92–298.70
AMCC	99.43	141.00 (69.00–295.00)	22.20–843.00	160.00 (82.20–347.50)	26.30–930.75	139.00 (67.73–288.00)	21.89–836.00
BMA	99.33	6.68 (3.43–13.00)	1.26–41.75	6.61 (3.38–12.88)	1.26–44.85	6.69 (3.44–13.00)	1.26–40.92
BPMA	75.32	3.75 (1.22–10.90)	0.85–48.53	2.77 (0.85–7.55)	0.85–28.01	3.89 (1.26–11.30)	0.85–50.30
CEMA	98.86	101.00 (49.90–191.00)	16.50–511.00	120.50 (62.00–235.00)	20.60–572.90	99.50 (48.53–186.00)	15.90–506.00
CYMA	86.77	1.73 (0.83–10.60)	0.35–290.00	1.73 (0.91–14.88)	0.35–346.75	1.73 (0.83–10.10)	0.35–285.60
DHBMA	99.87	316.00 (193.00–505.50)	64.35–900.00	361.00 (212.00–572.00)	77.36–985.00	311.00 (169.00–497.00)	62.69–897.10
2HPMA	94.59	32.00 (16.10–62.70)	3.75–214.00	31.80 (16.90–64.55)	6.18–216.00	32.00 (16.00–62.40)	3.75–213.90
3HPMA	99.51	250.00 (125.00–522.00)	43.80–1976	258.00 (129.00–558.00)	46.80–2140.00	249.00 (124.00–517.00)	43.64–1970.00
IPM3	83.71	3.78 (1.75–8.97)	0.85–78.26	4.82 (2.24–11.85)	0.85–96.25	3.71 (1.70–8.88)	0.85–73.85
MA	98.72	141.00 (77.30–240.00)	28.265–563.00	144.00 (80.60–234.00)	27.20–541.00	140.00 (76.80–240.00)	28.32–566.00
MHBMA3	96.95	5.21 (2.63–11.70)	0.84–59.90	5.97 (2.99–13.15)	0.98–64.71	5.16 (2.59–11.55)	0.81–59.55
PGA	95.49	195.00 (97.18–342.00)	14.94–59.90	208.00 (106.00–356.00)	23.90–743.00	193.00 (95.60–464.00)	13.80–696.70
HPMMA	99.96	240.00 (129.00–475.00)	45.90–2110.00	272.00 (150.00–582.00)	48.22–2250.00	237.00 (150.00–582.00)	45.60–2080.00

LLOD, lower limit of detection; IQR, interquartile range.

### 3.3 Urinary VOC metabolites (VOCs) and total and individual CVDs

The concentrations of VOC in urine of non-CVD and CVD subgroups adjusted by sampling weight were shown in Supplementary Table S2. The adjusted OR of CVD prevalence significantly increased (Table 3) when total urinary VOCs concentrations rose (p for trend = 0.025). According to the core model, those in the higher quartiles of total VOCs had adjusted OR (95% CI) of 1.01 (95% CI: 0.78 to 1.31), 1.26 (95% CI: 1.05 to 1.21) and 1.75 (95% CI:1.35 to 1.64) for total CVD. Additionally, we discovered that the prevalence of CVD was significantly and favorably correlated with urine levels of AAMA, CEMA, CYMA, 2HPMA, 3HPMA, and MHBMA3 (p for trend = 0.035, 0.034, 0.001, 0.025, 0.009, and 0.011, respectively) in core mode.

**Table 3:** Adjusted odds ratios for associations between the urinary VOCs and the presence of total CVD in NHANES.^a^

VOCs (urine, ng/ml)	Basic model		Core model		Extended model	
	aOR (95% CI)	p trend	aOR (95% CI)	p trend	aOR (95% CI)	p trend
Total mVOCs		0.003		0.025		0.049
Q1	1.00		1.00		1.00	
Q2	1.15 (0.92–1.44)		1.01 (0.78–1.31)		1.08 (0.82–1.42)	
Q3	1.31 (1.10–1.35)		1.26 (1.05–1.21)		1.25 (1.01–1.45)	
Q4	1.48 (1.18–1.85)		1.75 (1.35–1.64)		1.47 (1.05–2.05)	
2MHA		0.186		0.335		0.116
Q1	1.00		1.00		1.00	
Q2	1.02 (0.78, 1.34)		1.06 (0.76, 1.48)		1.15 (0.81, 1.64)	
Q3	0.87 (0.68, 1.10)		0.87 (0.64, 1.17)		0.94 (0.67, 1.32)	
Q4	1.19 (0.87, 1.64)		1.16 (0.82, 1.63)		1.35 (0.92, 1.98)	
3MHA + 4MHA		0.035		0.311		0.114
Q1	1.00		1.00		1.00	
Q2	1.07 (0.80, 1.44)		0.97 (0.69, 1.36)		1.10 (0.76, 1.59)	
Q3	1.15 (0.88, 1.51)		1.05 (0.76, 1.44)		1.24 (0.85, 1.80)	
Q4	1.36 (1.00, 1.85)		1.15 (0.79, 1.68)		1.42 (0.90, 2.26)	
AAMA		0.003		0.035		0.021
Q1	1.00		1.00		1.00	
Q2	0.96 (0.72, 1.29)		0.89 (0.67, 1.17)		0.97 (0.72, 1.29)	
Q3	1.03 (0.76, 1.39)		0.92 (0.66, 1.29)		1.08 (0.72, 1.63)	
Q4	1.51 (1.15, 1.98)		1.29 (0.97, 1.71)		1.54 (1.01, 2.35)	
AMCC		0.007		0.442		0.075
Q1	1.00		1.00		1.00	
Q2	0.99 (0.70, 1.40)		0.80 (0.53, 1.21)		0.83 (0.53, 1.29)	
Q3	0.88 (0.60, 1.31)		0.64 (0.42, 1.00)		0.70 (0.43, 1.13)	
Q4	1.29 (0.94, 1.78)		0.92 (0.64, 1.32)		1.08 (0.69, 1.71)	
BMA		0.574		0.842		0.747
Q1	1.00		1.00		1.00	
Q2	1.01 (0.73, 1.41)		0.93 (0.62, 1.37)		0.94 (0.63, 1.42)	
Q3	0.85 (0.65, 1.12)		0.84 (0.60, 1.18)		0.86 (0.61, 1.21)	
Q4	0.93 (0.70, 1.25)		0.94 (0.66, 1.34)		1.01 (0.70, 1.44)	
BPMA		0.061		0.285		0.271
Q1	1.00		1.00		1.00	
Q2	1.03 (0.77, 1.38)		1.06 (0.77, 1.47)		1.08 (0.76, 1.53)	
Q3	0.92 (0.67, 1.25)		0.82 (0.58, 1.16)		0.86 (0.60, 1.23)	
Q4	0.78 (0.57, 1.07)		0.85 (0.59, 1.23)		0.86 (0.59, 1.23)	
CEMA		0.001		0.034		0.004
Q1	1.00		1.00		1.00	
Q2	1.37 (1.06, 1.78)		0.92 (0.89, 1.78)		1.43 (1.03, 1.99)	
Q3	1.11 (0.84, 1.47)		1.25 (1.05, 1.34)		1.11 (0.76, 1.63)	
Q4	1.74 (1.29, 2.33)		1.36 (1.01, 1.83)		1.80 (1.27, 2.55)	
CYMA		<0.001		0.001		<0.001
Q1	1.00		1.00		1.00	
Q2	1.17 (0.91, 1.50)		1.04 (0.76, 1.41)		1.09 (0.79, 1.50)	
Q3	1.25 (0.90, 1.74)		1.20 (0.81, 1.78)		1.39 (1.21, 2.14)	
Q4	2.18 (1.61, 2.95)		1.83 (1.30, 2.57)		2.05 (1.40, 3.00)	
DHBMA		0.012		0.323		0.049
Q1	1.00		1.00		1.00	
Q2	1.28 (0.93, 1.76)		1.06 (0.76, 1.49)		1.14 (0.81, 1.62)	
Q3	1.04 (0.72, 1.50)		0.85 (0.56, 1.29)		1.03 (0.63, 1.67)	
Q4	1.52 (1.13, 2.05)		1.19 (0.85, 1.67)		1.64 (1.00, 2.67)	
2HPMA		0.004		0.025		0.014
Q1	1.00		1.00		1.00	
Q2	1.05 (0.77, 1.42)		0.97 (0.68, 1.40)		1.05 (0.71, 1.55)	
Q3	1.19 (0.89, 1.60)		1.13 (1.10, 1.58)		1.35 (0.93, 1.96)	
Q4	1.50 (1.14, 1.97)		1.37 (1.00, 1.88)		1.62 (1.08, 2.41)	
3HPMA		<0.001		0.009		0.002
Q1	1.00		1.00		1.00	
Q2	1.21 (0.89, 1.64)		1.05 (0.72, 1.52)		1.14 (0.75, 1.72)	
Q3	1.21 (0.92, 1.59)		0.99 (0.72, 1.37)		1.14 (0.82, 1.59)	
Q4	1.77 (1.33, 2.34)		1.43 (1.07, 1.90)		1.66 (1.20, 2.30)	
IPM3		<0.001		0.037		0.060
Q1	1.00		1.00		1.00	
Q2	0.86 (0.52, 1.43)		0.69 (0.30, 1.59)		0.66 (0.17, 2.64)	
Q3	1.24 (0.68, 2.27)		0.96 (0.40, 2.36)		1.01 (0.24, 4.31)	
Q4	1.82 (1.19, 2.78)		1.32 (1.21, 2.43)		1.33 (0.50, 3.56)	
Q1	1.00		1.00		1.00	
Q2	0.86 (0.52, 1.43)		0.90 (0.63, 1.30)		1.00 (0.68, 1.47)	
Q3	1.24 (0.68, 2.27)		0.97 (0.73, 1.29)		1.13 (0.82, 1.54)	
Q4	1.82 (1.19, 2.78)		1.05 (0.77, 1.44)		1.35 (0.87, 2.10)	
MHBMA3		<0.001		0.011		0.004
Q1	1.00		1.00		1.00	
Q2	0.94 (0.69, 1.28)		0.84 (0.57, 1.24)		0.89 (0.60, 1.32)	
Q3	1.05 (0.81, 1.37)		0.94 (0.67, 1.31)		1.05 (0.72, 1.52)	
Q4	1.74 (1.29, 2.35)		1.33 (1.14, 1.88)		1.51 (1.01, 2.25)	
PGA		0.601		0.630		0.636
Q1	1.00		1.00		1.00	
Q2	1.03 (0.75, 1.42)		0.94 (0.66, 1.33)		0.97 (0.67, 1.40)	
Q3	1.15 (0.82, 1.62)		0.99 (0.67, 1.44)		1.09 (0.74, 1.61)	
Q4	1.08 (0.77, 1.51)		0.91 (0.65, 1.28)		1.07 (0.74, 1.54)	
HPMMA		<0.001		0.141		0.089
Q1	1.00		1.00		1.00	
Q2	0.96 (0.70, 1.32)		0.92 (0.64, 1.32)		0.98 (0.67, 1.43)	
Q3	1.02 (0.74, 1.39)		0.91 (0.63, 1.31)		1.03 (0.69, 1.54)	
Q4	1.55 (1.18, 2.04)		1.13 (0.84, 1.53)		1.25 (0.88, 1.77)	

a Adjusted covariates: Basic model: age, sex, race, and NHANES cycles; Core model: basic model plus family PIR, education levels, physical activity levels, drinking, smoking status, BMI, diabetes, and hypertension; Extended model: core model plus serum total cholesterol, urine creatinine and urine albumin; CI: confidence interval. aOR: adjusted odds ratio. p trend, p for trend.

**Figure 3:**
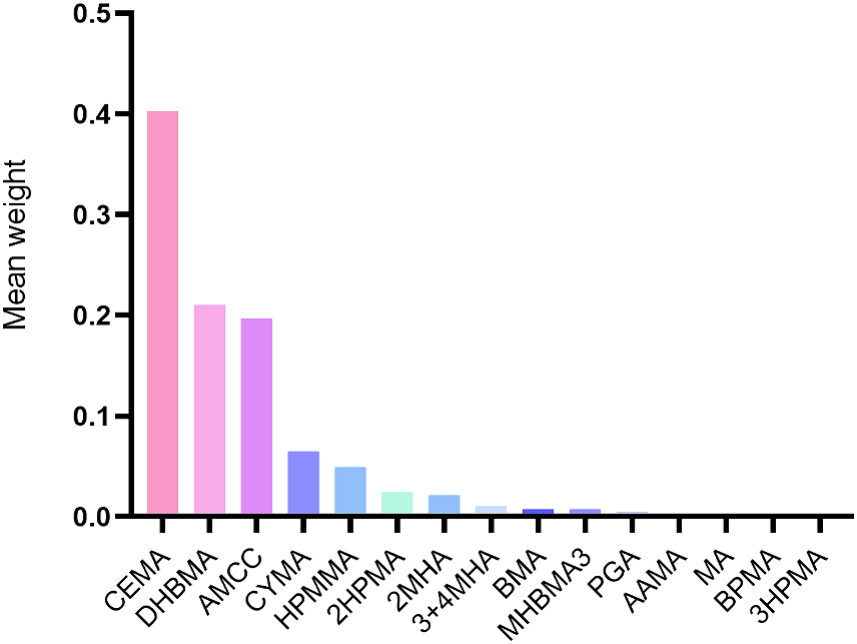
Weighted values of urinary VOCs for CVD in Weighted quantile sum models. Models were adjusted for covariates: age, sex, race, NHANES cycles, family PIR, education levels, physical activity levels, drinking or smoking status, body mass index, diabetes, hypertension, serum total cholesterol, urine creatinine and urine albumin.

The WQS index for mixed metals showed a positive association with CVD risk (OR 1.14, 95% CI 1.01 to 1.30). Furthermore, the most influential VOCs in the WQS models were CEMA (40.30%), DHBMA (21.00%), and AMCC (19.70%) (Figure 3).

**Figure 4:**
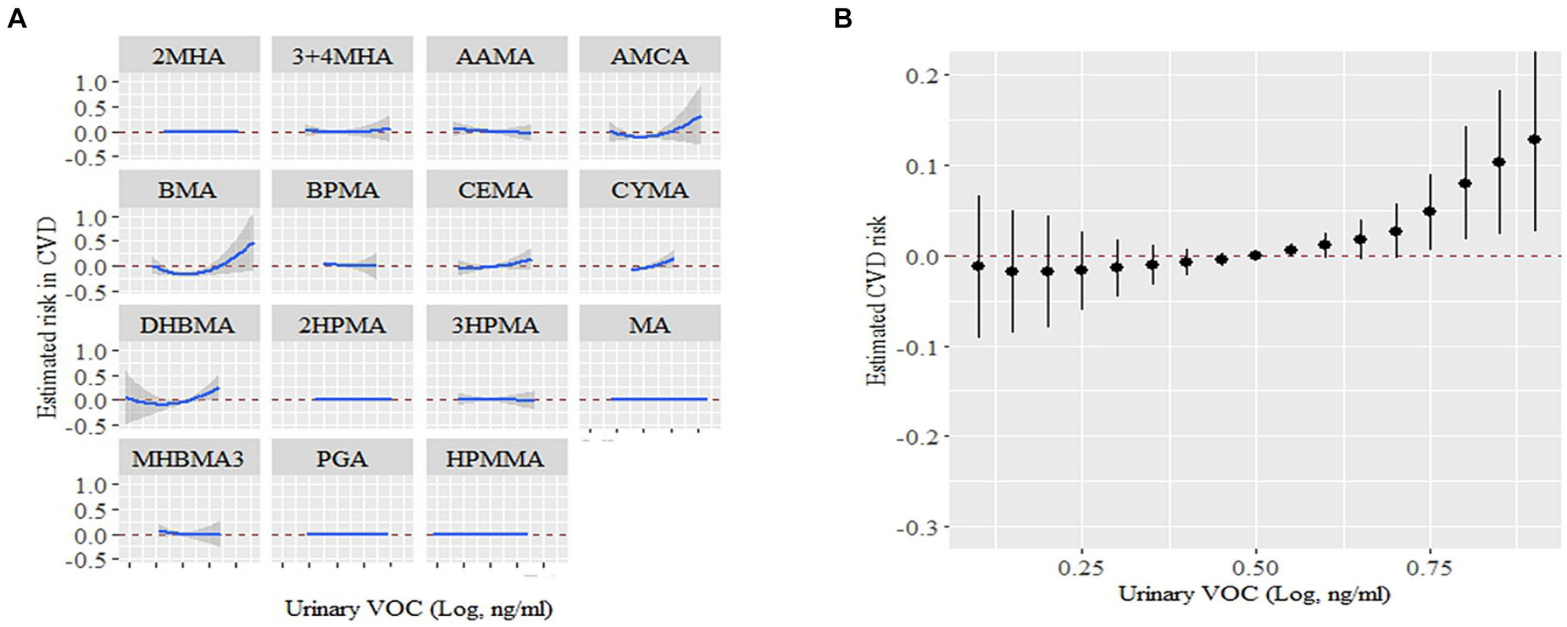
The links between urinary VOCs and the estimation of CVD risk using Bayesian Kernel Machine Regression (BKMR). **(A)** Exposure-response functions for each VOCs with the other VOCs fixed at the median. **(B)** Combined effects of urinary VOCs mixture on CVD risk. This graph depicted the estimated variance in CVD risk along with the corresponding 95% confidence interval when VOCs concentrations were maintained at specific percentiles in contrast to their respective medians. Models were adjusted for covariates: age, sex, race, NHANES cycles, family poverty income ratio, education levels, physical activity levels, drinking or smoking status, body mass index, diabetes, hypertension, serum total cholesterol, urine creatinine and urine albumin.

We also examined the cumulative and potential interaction effects of VOCs through the BKMR model. The BKMR model also showed a significant positive association between mixed VOCs and CVD. Figure 4A shows the trends in exposure-response functions for different kind of VOCs. AMCC, BMA, CEMA and CYMA showed increased associations with CVD when other VOCs concentrations were at their median levels. Figure 4B shows the overall correlation of a mix of VOCs on potential continuous outcomes, albeit with wide confidence intervals, when all chemical concentrations are in their 75th percentile or higher, relative to their 50th percentile Deciles, the underlying continuous outcome of CVD showed a significant increase, indicating a significant positive association with CVD.

**Figure 5:**
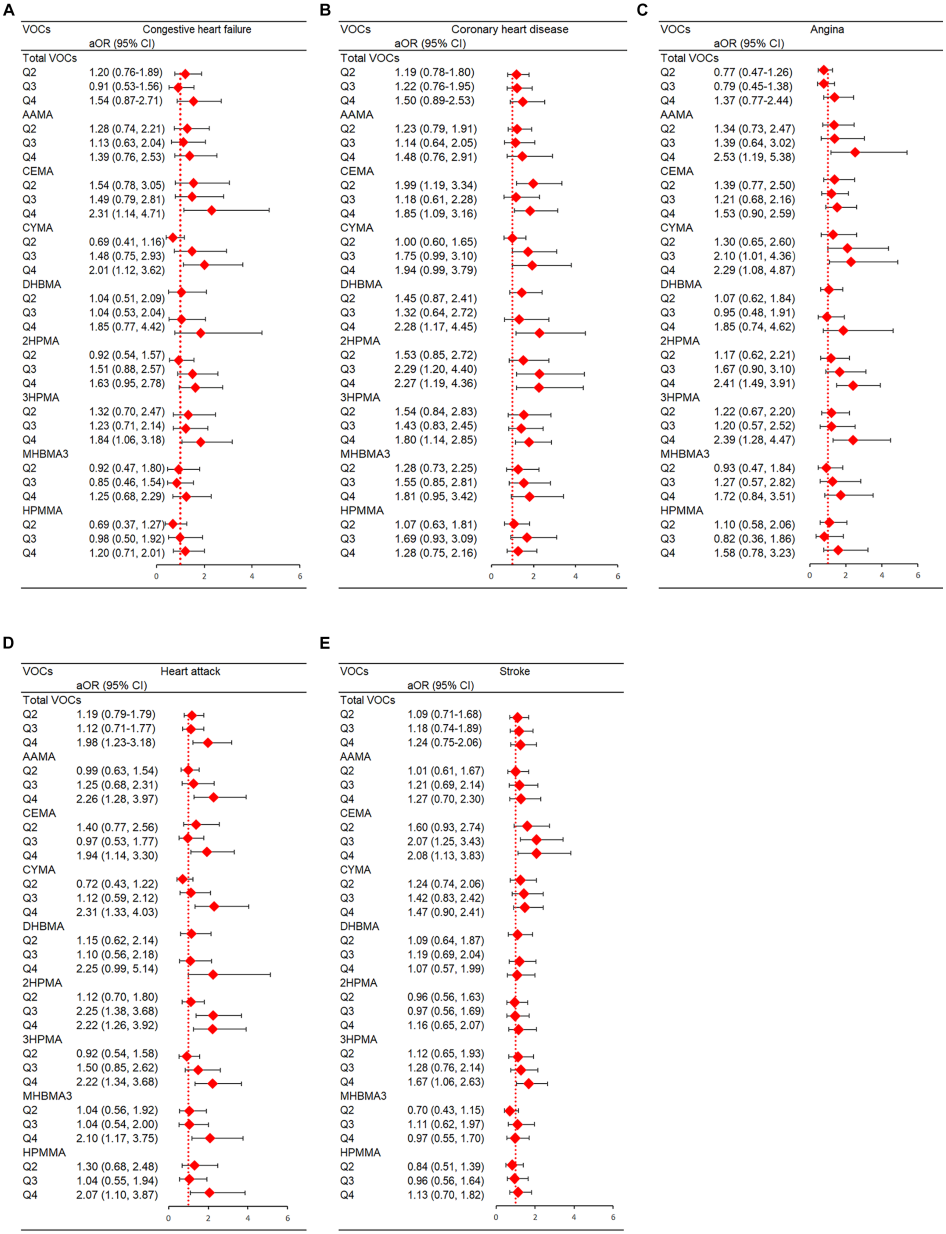
Statistically significant VOC and presence of individual CVDs in NHANES. **(A)** Congestive heart failure; **(B)** Coronary artery disease; **(C)** Angina; **(D)** Heart attack; **(E)** Stroke. Models were adjusted for covariates: age, sex, race, NHANES cycles, family PIR, education levels, physical activity levels, drinking or smoking status, body mass index, diabetes, hypertension, serum total cholesterol, urine creatinine and urine albumin.

Total VOCs had a strong correlation with heart attack in terms of the prevalence of individual CVDs (p for trend = 0.007). Urine levels of CYMA, CEMA, and 3HPMA were shown to be positively linked with congestive heart failure (p for trend = 0.005, 0.020, and 0.032, respectively) after complete adjustments. In addition, the prevalence of coronary heart disease was strongly correlated with DHBMA and 2HPMA (p for trend = 0.035, and 0.049 respectively); coronary heart disease was correlated with 3HPMA and CEMA at borderline meaningful values (p for trend = 0.070, and 0.230, respectively). AAMA, CYMA, 2HPMA, and 3HPMA were all positively related to angina (p for trends were 0.001, 0.009, and 0.001, respectively). A favorable association between heart attack and AAMA, CYMA, CEMA, DHBMA, 2HPMA, 3HPMA, MHBMA3, and HPMMA was found (p for trend = 0.007, <0.001, <0.001, 0.008, 0.032, 0.009, <0.001 < 0.001, and < 0.001, respectively). In addition, CEMA and 3HPMA were strongly correlated with stroke based on our study (p for trend = 0.045, and 0.016). In Figure 5, we show statistically significant VOCs and individual CVDs.

### 3.4 Subgroup analyses

With a rise in AAMA levels, those who were male had a BMI ≤25 kg/m^2^, smoked cigarettes, had a lower family PIR, actively used alcohol, and had a history of diabetes or hypertension had an increase the risk of CVD (Supplementary Table S4). People who were young, had a high family PIR and had a BMI ≤ 25 kg/m^2^, were more likely to suffer CVD when AMCC levels rose (Supplementary Table S5). People who were older adults, had a low family PIR, had a BMI ≤ 25 kg/m^2^ and had no history of diabetes and hypertension increased the risk of CVD when CEMA and DHBMA levels rose (Supplementary Tables S6, S7). Moreover, Male, low family PIR, BMI 25 ≤ kg/m^2^, drinking, and diabetes showed strong correlations between 3HPMA and CVD (Supplementary Table S8). Detailed information can be found in the Supplementary Tables S3–S9.

The results of our study indicated that VOC was found to have a significant association with CVD when comparing results from different models. These findings hold significant potential for public health implications and offer valuable insights for future research directions.

## 4 Discussion

In the current study, we mainly provided a novel finding that AAMA, CEMA, CYMA, 2HPMA, 3HPMA, IPM3, and MHBMA3 (acrolein, acrylamide, acrylonitrile, propylene oxide, isoprene, and 1,3-butadiene) were shown to be substantially and favorably linked with the prevalence of CVD based on the U.S. general population. The observed relationships were shown to be unaffected by many established risk factors for CVD, including age, sex, BMI, race, physical activity, smoking, alcohol intake, diabetes, kidney function, hypertension, and serum total cholesterol.

VOC are widely used in individuals’ everyday activities, encompassing car emissions, culinary practices, combustion of wood, diverse industrial operations, tobacco consumption, cleaning agents, construction materials, and numerous home commodities (19, 20). An increasing body of research has emerged indicating that VOC may be hazardous elements that have an impact on public health (16). Evidence from a previous study indicated that exposure to VOC can elevate serum CRP and 8-hydroxy-2′-deoxyguanosine levels and reduce heart rate variability indices (21). This is similar to our study, and we also discovered a favorable relationship between VOC exposure and CRP. Ye et al. found that emergency department visits for CVD are closely related to VOC exposure (22). Moreover, previous epidemiological researches have shown that CVD risk factors, including hypertension, blood glucose, diabetes, obesity, endothelial damage, and biomarkers indicative of oxidative stress, may be linked to exposure to VOC (7–11, 23). VOC have been discovered as an important factor in the development of hypertension, according to a study by McGraw et al. The research findings indicate that acrolein and styrene are the primary VOC responsible for hypertension in non-smokers, and crotonaldehyde is the primary VOC associated with hypertension in smokers (23). An additional study determined that exposure to VOC (acrolein and 1,3-butadiene) might lead to impairment of endothelial function and could heighten the likelihood of hypertension in persons with heightened sympathetic activity, particularly among those of black ethnicity (7). In addition, a recent NHANES study that included 1,409 participants revealed that exposure to VOC had a significant impact on insulin resistance and glucose homeostasis. This, in turn, has implications for the prevalence of diabetes, leading to notable public health concerns (8). The research has found that even low-level VOC exposure is linked to a reduction in circulating angiogenic cells, which might hamper endothelial repair and angiogenesis and increase the risk of CVD (9). Some studies have also suggested that exposure of VOC is closely related to obesity and dyslipidemia (10, 24).

However, few large-scale studies have directly reported that VOC exposure is closely related to CVD. Here, we have a thorough display of the links between 16 VOCs and CVD. In our study, Congestive heart failure was strongly correlated with CYMA, CEMA, and 3HPMA (acrylonitrile and acrolein) after all adjustments. Coronary heart disease was strongly correlated with DHBMA and 2HPMA (1,3-butadiene and propylene oxide). AAMA, CYMA, 2HPMA, and 3HPMA (acrylamide, acrylonitrile, propylene oxide, and acrolein) were positively associated with angina pectoris. Heart attack risk was strongly correlated with AAMA, CYMA, CEMA, DHBMA, 2HPMA, 3HPMA, MHBMA3, and HPMMA (acrylamide, acrylonitrile, acrolein, 1,3-butadiene, propylene oxide, and crotonaldehyde). In addition, CEMA and 3HPMA (acrolein) were strongly correlated with stroke based on our research. The mixed exposure is measured by WQS with an empirically weighted index in the direction of the joint action, in which components of concern are identified by non-negligible weights. This method truly reflects the joint action of mixed exposure. In our study, CEMA, DHBMA, and AMCC were identified as the strongest risk factors for CVD. Also, mixed VOCs were positively associated with CVD risk in WQS models and BKMR models, further suggesting that metal exposure may promote CVD progress.

Numerous studies have demonstrated that the interaction of oxidative stress with underlying biological components, which increases protein oxidation, lipid peroxidation, or DNA damage, results in the production of VOC (10). The human cardiovascular system is vulnerable to toxic substances. It has been suggested that breathing in particulate matter might cause lung oxidative stress and inflammation, which can then spread throughout the body and change how the central nervous system functions and raise the risk of cardiovascular events (25). This biological causation may also be used to explain how VOC exposure and CVD are related. A significant secondary pollutant, ozone (O3), is created when VOC from solvents or vehicle emissions are photochemically oxidized. Studies have demonstrated that exposure to high concentrations of O3 causes autonomic dysfunction in humans, as well as an inflammatory response *in vitro* cellular models and humans (21, 26). For example, the intake of elevated concentrations of ozone or volatile aldehydes has been found to induce cardiovascular and pulmonary harm activating the TRPA1 receptor in both humans and animals (27). Sensory nerves that have been activated provide signals to the central nervous system, resulting in the modification of the baroreceptor reflex, which is responsible for regulating blood pressure and maintaining appropriate cardiovascular function (28). In addition, Pal et al. found that the urinary VOCs were strongly positively linked with seven oxidative stress indicators (29).

Reactive oxygen species can be produced as a result of VOC exposure, and these species can then cause oxidative damage to biological macromolecules such proteins, lipids, and DNA (30). According to Chen et al. (11), several urinary VOCs were substantially linked to an increased risk of gestational diabetes mellitus in early pregnancy and may have been connected to indicators of oxidative stress. How oxidative stress affects the development of cardiovascular events as well as the initiation and progression of atherosclerosis has been extensively discussed (31, 32). Oxidative pathways, including enzymes such as NADPH oxidase, myeloperoxidase, superoxide dismutase, and glutathione peroxidase, play a major role in producing ROS (31). Specifically, NADPH oxidases and myeloperoxidase (MPO) are well-established enzymatic systems that play a crucial role in the progression of atherosclerosis by contributing to the formation of ROS (33).

In addition, there exists evidence indicating that the exposure to VOC (acrolein) has the potential to elevate the risk of CVD by inducing an elevation in blood pressure levels, as observed in both normotensive and hypertensive rat models (34), suppressing endothelial nitric oxide synthase activation (35), diminishing the migration of endothelial cells (36), obstructing the action of vascular endothelial growth factor (37), and decreasing the amounts of angiogenic cells in circulation, specifically in participants with hypertension (9). Several studies have also documented that exposure to 1,3-butadiene is associated with an elevated risk of CVD. Specifically, it has been found to increase the likelihood of high blood pressure in pregnant women with normal BP, impair endothelial function and raise diastolic blood pressure in individuals who do not smoke, and contribute to the development of arteriosclerotic heart disease in employees who are exposed to this chemical (38). Crotonaldehyde, a kind of VOC, is associated with the development of atherosclerosis, elevated blood pressure, and enhanced toxicity in the aorta and superior mesenteric artery, regardless of an individual’s smoking status (39, 40). We urge policymakers to prioritize monitoring VOC levels in the environment and in the bodies of high-risk individuals. Increased attention and stricter regulations are essential for controlling VOC concentrations. Media coverage can also help raise public awareness about VOCs, potentially leading to their routine inclusion in air quality reports as a significant pollutant.

The present study possesses some notable strengths, such as its utilization of a general population sample, its inclusion of individuals from diverse ethnic backgrounds, its large national sample size, and its inclusion of an adequate number of confounding variables for outcome adjustments. Three logistic regression models were employed to examine the data, and the study findings exhibited consistent stability across all three models. Moreover, these findings have substantial potential for public health implications and provide valuable insights for future research directions. However, it is essential to acknowledge some limitations that should not be disregarded. First, the data included in this cross-sectional investigation was obtained at once, hence limiting the ability to establish a causal association between VOC exposure and CVD. Second, it is important to acknowledge that the classification of health outcomes may be subject to misclassification due to the reliance on self-reported diagnoses for CVD outcomes. This introduces the possibility of memory bias, which might potentially impact the accuracy of the reported results. Third, while we made an effort to account for several potential risk factors in the statistical analyses, it is still important to proceed with caution when evaluating the association between adult CVD prevalence and VOCs exposure. This is because, in addition to VOCs, other confounders, such as genetic factors, may have also had an impact on CVD prevalence and should be taken into account in future research. It would be beneficial to propose future research designs that could address these limitations.

## 5 Conclusion

The results of our study indicated that VOC was all found to have a significant association with CVD when comparing results from different models. These findings hold significant potential for public health implications and offer valuable insights for future research directions. At the same time, we hope to call on more researchers and policymakers to pay attention to the problems we have discovered.

## Data availability statement

Publicly available datasets were analyzed in this study. This data can be found at: https://wwwn.cdc.gov/nchs/nhanes/Default.aspx.

## Ethics statement

The studies involving humans were approved by The study was conducted was approved by the Institutional Review Board of the National Centre for Health Statistics. The studies were conducted in accordance with the local legislation and institutional requirements. Written informed consent for participation in this study was provided by the participants' legal guardians/next of kin.

## Author contributions

SH: Data curation, Formal analysis, Methodology, Writing – original draft. MX: Data curation, Formal analysis, Writing – original draft. SC: Data curation, Writing – review & editing. YH: Software, Writing – original draft. PL: Software, Visualization, Writing – review & editing. JG: Funding acquisition, Project administration, Writing – review & editing.

## Footnotes

^1^ https://www.cdc.gov/nchs/nhanes/index.htm

^2^ https://wwwn.cdc.gov/nchs/data/nhanes/2015-2016/labmethods/UVOC_UVOCS_I_MET.pdf
