# Supplementary material for: Associations between specific volatile organic chemical exposures and cardiovascular disease risks: insights from NHANES
Source: Front Public Health. 2024 May 23;12:1378444. doi: 10.3389/fpubh.2024.1378444 (PMC11153666; doi:10.3389/fpubh.2024.1378444)
Supplement: Supplementary file 1 [file Table_1.DOCX]

# Supplemental Material

**Table S1.** Relative parent compounds to VOCs detected in this study.

| Parent Compound | VOC Metabolite | Common Name |
| --- | --- | --- |
| Acrolein | N-Acetyl-S-(2-carboxyethyl)-L-cysteine | CEMA |
|  | N-Acetyl-S-(3-hydroxypropyl)-L-cysteine | 3HPMA |
| Acrylamide | N-Acetyl-S-(2-carbamoylethyl)-L-cysteine | AAMA |
| Acrylonitrile | N-Acetyl-S-(2-cyanoethyl)-L-cysteine | CYMA |
| 1-Bromopropane | N-Acetyl-S-(n-propyl)-L-cysteine | BPMA |
| 1,3-Butadiene | N-Acetyl-S- (3,4-dihydroxybutyl)-L-cysteine | DHBMA |
|  | N-Acetyl-S-(4-hydroxy-2-buten-1-yl)-L-cysteine | MHBMA3 |
| Crotonaldehyde | N-Acetyl-S-(3-hydroxypropyl-1-methyl)-L-cysteine | HPMMA |
| N, N- Dimethylformamide | N-Acetyl-S-(N-methylcarbamoyl)-L-cysteine | AMCC |
| Ethylbenzene, styrene | Phenylglyoxylic acid | PGA |
| Propylene oxide | N-Acetyl-S-(2-hydroxypropyl)-L-cysteine | 2HPMA |
| Styrenea | Mandelic acid | MA |
| Isoprene | N-Acetyl- S- (4- hydroxy- 2- methyl- 2- butenyl)- L- cysteine | IPM3 |
| Toluenea | N-Acetyl-S-(benzyl)-L-cysteine | BMA |
| Xylenea | 2-Methylhippuric acid | 2MHA |
|  | 3-Methylhippuric acid + 4-Methylhippuric acid | 3MHA+4MHA |

**Table S2**. The concentrations of volatile organic compound metabolites (VOCs) in urine of non-CVD and CVD subgroups adjusted by sampling weight.

| VOCs (urine, ng/ml) | CVD (median ,IQR) | non-CVD (median ,IQR) | *p* |
| --- | --- | --- | --- |
| 2MHA | 29.30 (12.90, 75.30) | 26.67 (12.23, 74.72) | 0.326 |
| 3MHA+4MHA | 186.00 (78.50, 494.00) | 199.00 (84.99, 533.60) | 0.240 |
| AAMA | 50.40 (25.70, 100.00) | 49.40 (25.21, 101.00) | 0.839 |
| AMCA | 150.00 (70.90, 310.00) | 159.00 (80.00, 355.21) | 0.017 |
| BMA | 6.18 (3.19, 11.90) | 5.93 (3.29, 12.10) | 0.915 |
| BPMA | 3.61 (1.23, 10.40) | 2.66 (0.85, 7.18) | <0.001 |
| CEMA | 92.20 (43.90, 179.00) | 107.00 (56.45, 227.00) | <0.001 |
| CYMA | 1.62 (0.76, 9.05) | 1.69 (0.83, 14.88) | 0.184 |
| DHBMA | 302.00 (163.00, 497.00) | 338.00 (192.98, 570.45) | 0.001 |
| 2HPMA | 30.70 (15.40, 61.80) | 32.41 (16.04, 69.52) | 0.230 |
| 3HPMA | 235.00 (112.00, 501.00) | 241.00 (119.00, 532.54) | 0.430 |
| IPM3 | 3.51 (1.66, 8.24) | 5.07 (2.11, 12.30) | 0.005 |
| MA | 135.00 (71.60, 234.00) | 135.00 (71.67, 228.00) | 0.864 |
| MHBMA3 | 4.84 (2.40, 10.70) | 5.44 (2.66, 12.60) | 0.016 |
| PGA | 194.00 (98.30, 345.00) | 206.57 (105.00, 336.00) | 0.260 |
| HPMMA | 228.00 (116.00, 454.00) | 264.00 (136.00, 569.75) | 0.002 |

CVD: cardiovascular disease

**Table S3.** Subgroup analyses betweenc3MHA+4MHA and the presence of total CVD

| Subgroups | 3MHA+4MHA [aOR (95% CI)] | | | |
| --- | --- | --- | --- | --- |
|  | Q1 | Q2 | Q3 | Q4 |
| Age |  |  |  |  |
| ≤60 years | 1.00 | 1.21(0.73-2.03) | 1.71(1.04-2.82) | 1.79(1.06-3.04) |
| >60 years | 1.00 | 1.05(0.77-1.42) | 1.15(0.83-1.60) | 1.08(0.76-1.53) |
| Sex |  |  |  |  |
| Male | 1.00 | 1.07(0.74-1.54) | 1.18(0.81-1.72) | 1.29(0.87-1.91) |
| Female | 1.00 | 1.045(0.71-1.53) | 1.45(0.97-2.16) | 1.34(0.87-2.06) |
| Family PIR |  |  |  |  |
| Low | 1.00 | 1.06(0.78-1.46) | 1.40(1.01-1.93) | 1.45(1.04-2.03) |
| High | 1.00 | 1.21(0.74-1.95) | 1.13(0.67-1.91) | 1.21(0.69-2.14) |
| BMI |  |  |  |  |
| ≤25 kg/m^2^ | 1.00 | 1.34(0.74-2.42) | 2.24(1.26-3.99) | 1.94(1.06-3.57) |
| ＞25 kg/m^2^ | 1.00 | 1.07(0.80-1.44) | 1.14(0.83-1.55) | 1.25(0.90-1.74) |
| Smoking status |  |  |  |  |
| Yes | 1.00 | 1.13(0.78-1.63) | 1.23(0.85-1.79) | 1.36(0.94-1.97) |
| No | 1.00 | 1.03(0.70-1.50) | 1.41(0.93-2.13) | 1.19(0.72-1.96) |
| Alcohol |  |  |  |  |
| Yes | 1.00 | 1.24(0.81-1.89) | 1.45(0.92-2.28) | 1.76(1.08-2.84) |
| No | 1.00 | 1.04(0.74-1.45) | 1.25(0.86-1.76) | 1.26(0.88-1.81) |
| Hypertension |  |  |  |  |
| Yes | 1.00 | 0.93(0.68-1.27) | 1.14(0.81-1.59) | 1.276(0.90-1.90) |
| No | 1.00 | 1.77(1.09-2.87) | 1.99(1.20-3.28) | 1.719(1.00-2.95) |
| Diabetes |  |  |  |  |
| Yes | 1.00 | 0.90(0.58-1.39) | 1.05(0.64-1.72) | 1.44(0.84-2.47) |
| No | 1.00 | 1.21(0.87-1.67) | 1.45(1.04-2.01) | 1.33(0.942-1.88) |

Adjusted covariates: age, sex, race/ethnicity, NHANES cycles, family PIR, education levels, physical activity levels, drinking, smoking status, BMI, diabetes, hypertension, serum total cholesterol, urine creatinine and urine albumin; aOR: adjusted odds ratio.

**Table S4.** Subgroup analyses between AAMA and the presence of total CVD

| Subgroups | AAMA [aOR (95% CI)] | | | |
| --- | --- | --- | --- | --- |
|  | Q1 | Q2 | Q3 | Q4 |
| Age |  |  |  |  |
| ≤60 years | 1.00 | 1.15(0.71-1.87) | 1.41(0.85-2.32) | 1.38(0.80-2.32) |
| >60 years | 1.00 | 0.95(0.70-1.28) | 1.05(0.75-1.45) | 1.41(0.96-2.07) |
| Sex |  |  |  |  |
| Male | 1.00 | 0.91(0.64-1.30) | 0.95(0.65-1.38) | 1.51(1.01-2.25) |
| Female | 1.00 | 0.97(0.67-1.42) | 1.40(0.94-2.09) | 1.34(0.82-2.17) |
| Family PIR |  |  |  |  |
| Low | 1.00 | 1.03(0.76-1.39) | 1.13(0.81-1.57) | 1.031(1.02-1.05) |
| High | 1.00 | 0.93(0.57-1.51) | 1.27(0.77-2.12) | 1.266(0.69-2.34) |
| BMI |  |  |  |  |
| ≤25 kg/m^2^ | 1.00 | 1.18(0.69-2.02) | 1.26(0.71-2.25) | 2.08(1.10-3.94) |
| ＞25 kg/m^2^ | 1.00 | 0.95(0.71-1.27) | 1.16(0.85-1.58) | 1.37(0.97-1.95) |
| Smoking status |  |  |  |  |
| Yes | 1.00 | 1.10(0.77-1.57) | 1.12(0.77-1.62) | 1.60(1.08-2.37) |
| No | 1.00 | 0.86(0.59-1.27) | 1.28(0.84-1.95) | 1.05(0.606-1.81) |
| Alcohol |  |  |  |  |
| Yes | 1.00 | 1.15(0.83-1.59) | 1.24(0.88-1.74) | 1.68(1.15-2.46) |
| No | 1.00 | 0.82(0.53-1.25) | 1.13(0.71-1.78) | 1.35(0.79-2.29) |
| Hypertension |  |  |  |  |
| Yes | 1.00 | 1.02(0.75-1.39) | 1.21(0.87-1.68) | 1.48(1.016-2.15) |
| No | 1.00 | 0.90(0.56-1.44) | 1.06(0.65-1.73) | 1.43(0.834-2.46) |
| Diabetes |  |  |  |  |
| Yes | 1.00 | 1.04(0.65-1.66) | 1.33(0.81-2.20) | 2.16(1.23-3.78) |
| No | 1.00 | 1.01(0.74-1.37) | 1.12(0.80-1.55) | 1.27(0.88-1.84) |

Adjusted covariates: age, sex, race/ethnicity, NHANES cycles, family PIR, education levels, physical activity levels, drinking, smoking status, BMI, diabetes, hypertension, serum total cholesterol, urine creatinine and urine albumin; aOR: adjusted odds ratio.

**Table S5.** Subgroup analyses between AMCC and the presence of total CVD

| Subgroups | Total AMCC [aOR (95% CI)] | | | |
| --- | --- | --- | --- | --- |
|  | Q1 | Q2 | Q3 | Q4 |
| Age |  |  |  |  |
| ≤60 years | 1.00 | 1.44(0.82-2.53) | 1.58(0.89-2.81) | 2.19(1.20-3.99) |
| >60 years | 1.00 | 0.84(0.60-1.19) | 0.78(0.54-1.11) | 1.08(0.73-1.61) |
| Sex |  |  |  |  |
| Male | 1.00 | 1.09(0.72-1.66) | 1.01(0.65-1.55) | 1.37(0.87-2.17) |
| Female | 1.00 | 0.76(0.50-1.14) | 0.79(0.51-1.22) | 1.43(0.89-2.28) |
| Family PIR |  |  |  |  |
| Low | 1.00 | 0.77(0.54-1.09) | 0.82(0.57-1.17) | 1.24(0.85-1.82) |
| High | 1.00 | 1.49(0.87-2.57) | 1.05(0.58-1.90) | 1.92(1.01-1.04) |
| BMI |  |  |  |  |
| ≤25 kg/m^2^ | 1.00 | 0.93(0.49-1.76) | 1.29(0.68-2.45) | 2.666(1.35-5.27) |
| ＞25 kg/m^2^ | 1.00 | 0.94(0.68-1.31) | 0.83(0.59-1.18) | 1.198(0.82-1.74) |
| Smoking status |  |  |  |  |
| Yes | 1.00 | 0.94(0.61-1.46) | 0.89(0.58-1.36) | 1.22(0.79-1.90) |
| No | 1.00 | 0.89(0.59-1.33) | 0.88(0.55-1.39) | 1.51(0.89-2.55) |
| Alcohol |  |  |  |  |
| Yes | 1.00 | 0.89(0.61-1.30) | 0.92(0.63-1.36) | 1.37(0.91-2.08) |
| No | 1.00 | 1.07(0.67-1.70) | 0.92(0.56-1.51) | 1.53(0.90-2.62) |
| Hypertension |  |  |  |  |
| Yes | 1.00 | 0.94(0.66-1.34) | 0.90(0.62-1.30) | 1.39(0.93-2.06) |
| No | 1.00 | 0.84(0.50-1.43) | 0.90(0.53-1.53) | 1.28(0.73-2.24) |
| Diabetes |  |  |  |  |
| Yes | 1.00 | 0.88(0.50-1.55) | 0.838(0.47-1.49) | 1.61(0.85-3.05) |
| No | 1.00 | 0.95(0.67-1.33) | 0.906(0.63-1.30) | 1.29(0.88-1.88) |

Adjusted covariates: age, sex, race/ethnicity, NHANES cycles, family PIR, education levels, physical activity levels, drinking, smoking status, BMI, diabetes, hypertension, serum total cholesterol, urine creatinine and urine albumin; aOR: adjusted odds ratio.

**Table S6**. Subgroup analyses between CEMA and the presence of total CVD

| Subgroups | CEMA [aOR (95% CI)] | | | |
| --- | --- | --- | --- | --- |
|  | Q1 | Q2 | Q3 | Q4 |
| Age |  |  |  |  |
| ≤60 years | 1.00 | 1.07(0.67-1.73) | 1.21(0.74-1.99) | 1.54(0.92-2.57) |
| >60 years | 1.00 | 1.16(0.84-1.60) | 1.03(0.73-1.45) | 1.54(1.00-2.20) |
| Sex |  |  |  |  |
| Male | 1.00 | 1.32(0.90-1.95) | 1.244(0.83-1.87) | 1.755(1.14-2.69) |
| Female | 1.00 | 0.91(0.62-1.33) | 0.895(0.59-1.35) | 1.309(0.85-2.02) |
| Family PIR |  |  |  |  |
| Low | 1.00 | 1.26(0.91-1.74) | 1.20(0.86-1.68) | 1.59(1.11-2.27) |
| High | 1.00 | 0.81(0.52-1.41) | 0.91(0.53-1.57) | 1.54(0.86-2.76) |
| BMI |  |  |  |  |
| ≤25 kg/m^2^ | 1.00 | 1.22(0.69-2.15) | 1.599(0.87-2.94) | 2.77(1.50-5.09) |
| ＞25 kg/m^2^ | 1.00 | 1.07(0.79-1.46) | 0.974(0.70-1.35) | 1.28(0.90-1.89) |
| Smoking status |  |  |  |  |
| Yes | 1.00 | 1.42(0.98-2.06) | 1.39(0.95-2.03) | 1.71(1.15-2.53) |
| No | 1.00 | 0.82(0.55-1.22) | 0.74(0.47-1.16) | 1.24(0.75-2.04) |
| Alcohol |  |  |  |  |
| Yes | 1.00 | 1.17(0.84-1.64) | 1.08(0.76-1.54) | 1.44(0.99-2.09) |
| No | 1.00 | 1.09(0.70-1.69) | 1.08(0.67-1.74) | 1.85(1.10-3.10) |
| Hypertension |  |  |  |  |
| Yes | 1.00 | 1.05(0.76-1.46) | 1.01(0.72-1.43) | 1.29(0.89-1.86) |
| No | 1.00 | 1.27(0.79-2.04) | 1.16(0.69-1.93) | 2.12(1.26-3.56） |
| Diabetes |  |  |  |  |
| Yes | 1.00 | 1.12(0.69-1.82) | 0.97(0.51-1.49) | 1.51(0.87-2.65) |
| No | 1.00 | 1.09(0.79-1.50) | 1.15(0.82-1.61) | 1.47(1.03-2.11) |

Adjusted covariates: age, sex, race/ethnicity, NHANES cycles, family PIR, education levels, physical activity levels, drinking, smoking status, BMI, diabetes, hypertension, serum total cholesterol, urine creatinine and urine albumin; aOR: adjusted odds ratio.

**Table S7.** Subgroup analyses between DHBMA and the presence of total CVD

| Subgroups | Total DHBMA[aOR (95% CI)] | | | |
| --- | --- | --- | --- | --- |
|  | Q1 | Q2 | Q3 | Q4 |
| Age |  |  |  |  |
| ≤60 years | 1.00 | 1.03(0.64-1.64) | 1.47(0.89-2.42) | 1.73(0.96-3.11) |
| >60 years | 1.00 | 1.22(0.87-1.71) | 1.14(0.78-1.67) | 2.49(1.57-3.96) |
| Sex |  |  |  |  |
| Male | 1.00 | 1.03(0.70-1.52) | 0.98(0.65-1.49) | 1.55(0.95-2.52) |
| Female | 1.00 | 1.11(0.75-1.66) | 1.31(0.83-2.07) | 2.61(1.50-4.55) |
| Family PIR |  |  |  |  |
| Low | 1 | 1.13(0.81-1.57) | 1.28(0.89-1.82) | 2.00(1.30-3.07) |
| High | 1 | 1.07(0.65-1.75) | 0.92(0.50-1.69) | 1.90(0.95-3.80) |
| BMI |  |  |  |  |
| ≤25 kg/m^2^ | 1 | 1.22(0.68-2.17) | 1.37(0.70-2.69) | 3.90(1.86-8.20) |
| ＞25 kg/m^2^ | 1 | 1.08(0.79-1.48) | 1.06(0.75-1.50) | 1.59(1.05-1.06) |
| Smoking status |  |  |  |  |
| Yes | 1.00 | 1.20(0.84-1.72) | 1.07(0.73-1.58) | 1.59(1.01-2.49) |
| No | 1.00 | 0.97(0.63-1.50) | 1.28(0.78-2.11) | 2.77(1.49-5.18) |
| Alcohol |  |  |  |  |
| Yes | 1 | 1.13(0.71-1.81) | 1.393(0.824-2.357) | 2.943(1.553-5.577) |
| No | 1 | 1.12(0.80-1.57) | 1.058(0.726-1.541) | 1.670(1.070-2.608) |
| Hypertension |  |  |  |  |
| Yes | 1.00 | 1.09(0.78-1.51) | 1.20(0.83-1.73) | 1.630(1.05-2.53) |
| No | 1.00 | 1.09(0.66-1.80) | 0.99(0.56-1.73) | 2.827(1.49-5.36) |
| Diabetes |  |  |  |  |
| Yes | 1.00 | 0.99(0.59-1.68) | 1.33(0.76-2.34) | 2.75(1.41-5.37) |
| No | 1.00 | 1.13(0.82-1.56) | 1.08(0.75-1.55) | 1.66(1.08-2.57) |

Adjusted covariates: age, sex, race/ethnicity, NHANES cycles, family PIR, education levels, physical activity levels, drinking, smoking status, BMI, diabetes, hypertension, serum total cholesterol, urine creatinine and urine albumin; aOR: adjusted odds ratio.

**Table S8.** Subgroup analyses between 3HPMA and the presence of total CVD

| Subgroups | 3HPMA [aOR (95% CI)] | | | |
| --- | --- | --- | --- | --- |
|  | Q1 | Q2 | Q3 | Q4 |
| Age |  |  |  |  |
| ≤60 years | 1.00 | 1.01(0.62-1.64) | 0.94(0.57-1.57) | 1.570(0.96-2.57) |
| >60 years | 1.00 | 0.88(0.65-1.20) | 1.09(0.78-1.53) | 1.20(0.83-1.72) |
| Sex |  |  |  |  |
| Male | 1.00 | 0.99(0.69-1.45) | 1.06(0.72-1.57) | 1.61(1.07-2.40) |
| Female | 1.00 | 0.79(0.54-1.15) | 1.07(0.71-1.62) | 1.33(0.87-2.04) |
| Family PIR |  |  |  |  |
| Low | 1.00 | 0.93(0.68-1.27) | 1.10(0.79-1.53) | 1.54(1.09-2.16) |
| High | 1.00 | 1.00(0.62-1.62) | 1.14(0.67-1.95) | 1.40(0.80-2.48) |
| BMI |  |  |  |  |
| ≤25 kg/m^2^ | 1.00 | 1.14(0.65-2.00) | 1.61(0.88-2.95) | 1.95(1.09-3.51) |
| ＞25 kg/m^2^ | 1.00 | 0.92(0.69-1.24) | 1.02(0.74-1.40) | 1.44(1.03-2.02) |
| Smoking status |  |  |  |  |
| Yes | 1.00 | 0.93(0.64-1.34) | 1.13(0.77-1.64) | 1.58(1.10-2.29) |
| No | 1.00 | 0.92(0.63-1.35) | 0.95(0.61-1.47) | 1.01(0.59-1.76) |
| Alcohol |  |  |  |  |
| Yes | 1.00 | 0.97(0.70-1.35) | 1.114(0.786-1.58) | 1.43(0.99-2.06) |
| No | 1.00 | 0.89(0.580-1.36) | 0.998(0.620-1.61) | 1.77(1.08-2.88) |
| Hypertension |  |  |  |  |
| Yes | 1.00 | 0.92(0.68-1.26) | 1.06(0.76-1.49) | 1.35(0.95-1.92) |
| No | 1.00 | 0.99(0.61-1.62) | 1.21(0.73-2.00) | 1.99(1.15-3.20) |
| Diabetes |  |  |  |  |
| Yes | 1.00 | 0.82(0.51-1.32) | 0.79(0.47-1.34) | 1.41(0.81-2.45) |
| No | 1.00 | 0.99(0.72-1.36) | 1.24(0.89-1.73) | 1.49(1.06-2.10) |

Adjusted covariates: age, sex, race/ethnicity, NHANES cycles, family PIR, education levels, physical activity levels, drinking, smoking status, BMI, diabetes, hypertension, serum total cholesterol, urine creatinine and urine albumin; aOR: adjusted odds ratio.

**Table S9. Subgroup analyses between total VOCs and the presence of total CVD in NHANES^a^**

| Subgroups | Total VOCs [aOR (95% CI)] | | | |
| --- | --- | --- | --- | --- |
|  | Q1 | Q2 | Q3 | Q4 |
| Age |  |  |  |  |
| ≤60 | 1.00 | 0.99(0.49-2.03) | 1.56(0.82-2.96) | 1.86(1.00-3.46)^*^ |
| >60 | 1.00 | 1.12(0.87-1.43) | 0.90(0.70-1.17) | 1.21(0.94-1.56) |
| Sex |  |  |  |  |
| Male | 1.00 | 0.97(0.66-1.43) | 0.70(0.45-1.08) | 1.14(0.72-1.79) |
| Female | 1.00 | 1.04(0.691-1.57) | 1.58(1.01-2.47) | 1.80(1.07-3.03)^*^ |
| Family PIR |  |  |  |  |
| Low | 1.00 | 1.68(0.59-4.76) | 1.77(0.54-5.84) | 1.83(0.53-6.25) |
| High | 1.00 | 0.99(0.60-1.66) | 0.89(0.49-1.61) | 1.14(0.56-2.30) |
| BMI |  |  |  |  |
| ≤25 kg/m^2^ | 1.00 | 1.19(0.65-2.17) | 1.35(0.704-2.58) | 2.00(1.02-3.94)^*^ |
| ＞25 kg/m^2^ | 1.00 | 1.05(0.76-1.44) | 1.00(0.698-1.42) | 1.32(0.90-1.95) |
| Smoking status |  |  |  |  |
| Yes | 1.00 | 1.34(0.90-2.00) | 1.23(0.81-1.86) | 1.657(1.08-2.54)^*^ |
| No | 1.00 | 0.80(0.53-1.21) | 0.84(0.51-1.38) | 0.961(0.50-1.85) |
| Alcohol |  |  |  |  |
| Yes | 1.00 | 1.01(0.71-1.44) | 0.92(0.63-1.35) | 1.23(0.81-1.87) |
| No | 1.00 | 1.23(0.79-1.93) | 1.38(0.81-2.34) | 2.11(1.19-3.76)^*^ |
| Hypertension |  |  |  |  |
| Yes | 1.00 | 1.10(0.78-1.54) | 1.10(0.76-1.61) | 1.49(0.98-2.25) |
| No | 1.00 | 1.02(0.62-1.69) | 1.01(0.58-1.76) | 1.40(0.78-2.52) |
| Diabetes |  |  |  |  |
| Yes | 1.00 | 0.85(0.50-1.42) | 0.83(0.46-1.51) | 2.02(1.05-3.88)^*^ |
| No | 1.00 | 1.23(0.88-1.71) | 1.20(0.83-1.73) | 1.26(0.84-1.88) |

a Adjusted covariates: age, sex, race, NHANES cycles, family PIR, education levels, physical activity levels, drinking or smoking status, BMI, diabetes, hypertension, serum total cholesterol, urine creatinine and urine albumin; The total VOCs levels were used for quartiles in study population: < 1290.17 ng/mL (quartile 1), 1290.17–2260.77 ng/mL (quartile 2), 2260.77–3753.67 ng/mL (quartile 3), and > 3753.66 ng/mL (quartile 4). CI: confidence interval. aOR: adjusted odds ratio; * P<0.05.
